# Improving access to emergency obstetric care in underserved rural Tanzania: a prospective cohort study

**DOI:** 10.1186/s12884-022-04951-1

**Published:** 2022-08-17

**Authors:** Angelo S. Nyamtema, Heather Scott, John C. LeBlanc, Elias Kweyamba, Janet Bulemela, Allan Shayo, Omary Kilume, Zabron Abel, Godfrey Mtey

**Affiliations:** 1 Tanzanian Training Centre for International Health, P.O Box 39, Ifakara, Tanzania; 2 Department of Obstetrics and Gynaecology, St. Francis University College for Health and Allied Sciences, Ifakara, Tanzania; 3 Department of Obstetrics and Gynaecology, Dalhousie University, Halifax, Canada; 4 Pediatrics, Community Health and Epidemiology and Psychiatry, Dalhousie University, Halifax, Canada; 5 Department of Paediatrics, St. Francis University College for Health and Allied Sciences, Ifakara, Tanzania

**Keywords:** Births in emergency obstetric care facility, Met need for emergency obstetric care, Case fatality rate, Tanzania

## Abstract

**Background:**

One of the key strategies to reducing maternal mortality is provision of emergency obstetric care services. This paper describes the results of improving availability of, and access to emergency obstetric care services in underserved rural Tanzania using associate clinicians.

**Methods:**

A prospective cohort study of emergency obstetric care was implemented in seven health centres in Morogoro region, Tanzania from July 2016 to June 2019. In early 2016, forty-two associate clinicians from five health centres were trained in teams for three months in emergency obstetric care, newborn care and anaesthesia. Two health centres were unexposed to the intervention and served as controls. Following training, virtual teleconsultation, quarterly on-site supportive supervision and continuous mentorship were implemented to reinforce skills and knowledge.

**Results:**

The met need for emergency obstetric care increased significantly from 45% (459/1025) at baseline (July 2014 – June 2016) to 119% (2010/1691) during the intervention period (Jul 2016 – June 2019). The met need for emergency obstetric care in the control group also increased from 53% (95% CI 49–58%) to 77% (95% CI 74–80%). Forty maternal deaths occurred during the baseline and intervention periods in the control and intervention health centres. The direct obstetric case fatality rate decreased slightly from 1.5% (95% CI 0.6–3.1%) to 1.1% (95% CI 0.7–1.6%) in the intervention group and from 3.3% (95% CI 1.2–7.0%) to 0.8% (95% CI 0.2–1.7%) in the control group.

**Conclusions:**

When emergency obstetric care services are made available the proportion of obstetric complications treated in the facilities increases. However, the effort to scale up emergency obstetric care services in underserved rural areas should be accompanied by strategies to reinforce skills and the referral system.

## Background

Maternal mortality is decreasing globally, but not fast enough and with critical inequalities across many regions of the world [1]. Provision of emergency obstetric and newborn care (EmONC) for prompt and appropriate management of complications that arise during pregnancy and childbirth is key to reducing maternal and perinatal mortality [2–4]. The government of Tanzania through its National Road Map Strategic Plan (One Plan II) for 2016–2020, set a goal to reduce maternal mortality from 556 in 2015 to 292 per 100,000 live births by 2020 [5]. One of the key strategies was to improve availability of, and accessibility to, comprehensive emergency obstetric care services [5, 6]. In countries with acute shortages of human resources for health, like Tanzania, scale up of comprehensive emergency obstetric and newborn care (CEmONC) may only be feasible through task-sharing, such as upgrading the skills of associate clinicians to perform obstetric and anaesthetic procedures normally done by specialist clinicians.

For several decades associate clinicians have formed the backbone of the health system in many resource-limited countries particularly in sub-Saharan countries [7]. In the context of high maternal and perinatal mortality and from a human rights perspective, provision of emergency obstetric surgical interventions and anaesthesia can be delegated to associate clinicians [8]. Evidence gathered from previous studies indicate no significant differences between the obstetric operations done by associate clinicians and trained physicians (medical officers) in either outcomes or quality of care indicators [9–11]. Projections indicate that if all women with complicated deliveries could reach health facilities where these providers operate, maternal mortality could fall by 75% or more [9]. In the context of a serious shortage of hospitals equipped to provide CEmONC, the government of Tanzania set targets to upgrade and scale up CEmONC services from 12% in 2015 to 75% of all public health centres by 2020 [5].

As part of the Innovating for Maternal and Child Health in Africa (IMCHA) initiative, an implementation research project (Accessing Safe Deliveries in Tanzania or ASDIT) was designed to study how to scale up accessibility to CEmONC services in underserved rural Tanzania using available human resources for health at the facilities. This paper provides information on the processes, and the impact of introducing and improving access to obstetric and newbornservices at health centres in underserved rural Tanzania. Specifically, measurable impact indicators were the proportion of expected births delivering in CEmONC facilities, the met need for emergency obstetric care and direct obstetric case fatality rate. ‘Met need’ is an estimate of the proportion of all women with major direct obstetric complications who are treated in a health facility providing EmONC. The direct obstetric case fatality rate is the proportion of women admitted to an emergency obstetric care facility with major direct obstetric complications, or who develop such complications after admission, and die before discharge [4].

## Materials and methods

### Settings

**Fig. 1:**
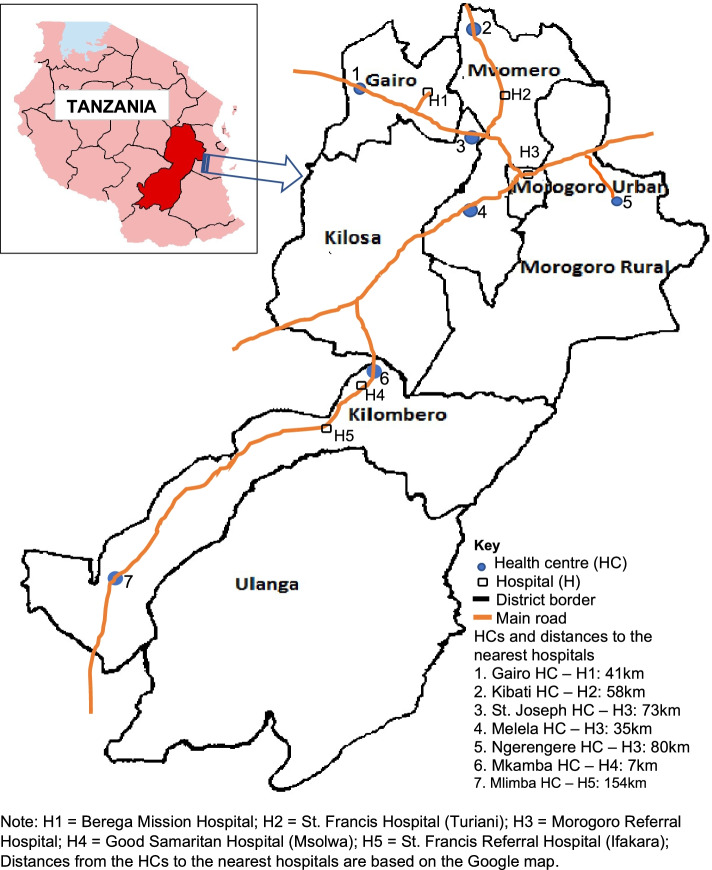
Map of Morogoro region indicating the geographical locations of the project health centres and the nearest hospitals

This was a prospective cohort study of CEmONC implementation in five health centres chosen because they were far from the nearest hospital and represented the different funding and governance models for health centres in Tanzania (Fig. 1). As reported elsewhere, Morogoro region had 15 health centres that were either already offering CEmONC or were ready to do so once staff were trained [12]. The first category included three publicly-funded HCs that had never provided CEmONC services. They had the proper infrastructure (maternity and neonatal wards, a functioning operating theatre and ability to provide emergency blood transfusions) but their staff had not received CEmONC training. This group typified the HCs that the Ministry of Health would have to upgrade as it implements its national goal of 50% of health centres in Tanzania offering CEmONC. Two of the three (Kibati and Ngerengere) HCs were randomly allocated to the intervention. The second category had nine publicly-funded HCs and were already providing CEmONC. Using simple random sampling, two of the nine (Mlimba and Mkamba HCs) were allocated to be control sites and two (Gairo and Melela HCs) to the intervention in order to study how CEmONC services could be strengthened. The third category contained three HCs affiliated with faith-based organizations. They receive both public and faith-based organization funding and are a permanent and integral part of the Tanzanian health system. One of the three (St. Joseph HC) was randomly allocated to the intervention. Unlike many studies where control centres are chosen to be comparable to intervention centres, we chose two facilities primarily to track secular trends, i.e., changes in epidemiology and health services practices that occurred independently of the ASDIT intervention. Again, unlike many studies, we purposefully chose our five intervention HCs to be different from each other in order to understand the variation in experience according to how facilities were funded and administered. With the exception of Kilosa district, none had ready access to a district hospital. Before the intervention, women with emergency obstetric complications requiring surgical intervention in the four health centres were referred to either a nearby faith-based hospital or a regional referral hospital in Morogoro urban. The distance from these health centres to the nearest district hospital ranged from 35 km to 80 km.

### Interventions

Face-to-face training in CEmONC and anaesthesia: Twenty six associate clinicians from five health centres were trained in teams for three months in CEmONC and anaesthesia. From each health centre, four to six associate clinicians were trained. Assistant medical officers (advanced associate clinicians) were trained in CEmONC while clinical officers (associate clinicians) and nurse-midwives (ordinary diploma holders) were trained in anaesthesia and postoperative care of mothers and newborns. In Tanzania, clinical officers are mid-level professionals trained in a three-year post-secondary clinical medicine program and are not licensed to perform major surgery. Assistant medical officers are clinical officers with an additional two-year training program in clinical medicine, which includes three months of surgery and three months of obstetrics. They function as general practitioners and are licensed to perform major surgery independently, including caesarean sections. Although both often lack hands-on experience in surgery and obstetrics at the time of graduation, university graduate medical doctors go through a one-year internship training whereas assistant medical doctors do not. The project team adopted and revised the CEmONC and anaesthesia training curricula designed at Tanzanian Training Centre for International Health (TTCIH) and St. Francis Referral Hospital (SFRH) described elsewhere [13]. The main emphasis of both training curricula included the use of underlying principles in obstetric and anaesthetic care; appropriate decision making and clinical reasoning skills, and acquisition of clinical management skills in these areas. Both training programs were full time and took place at SFRH, a busy facility, to enhance hands-on practice and acquisition of skills. The educational curricula were implemented by teams of obstetricians, pediatricians and anaesthetists working at SFRH and TTCIH.

Post-training capacity building: Following training, the research team implemented teleconsultation, quarterly on-site supportive supervision and continuous mentorship via telephone and social media to reinforce skills and knowledge. Care providers at the health centres were linked to obstetricians for virtual consultation when there were maternal complications. Supportive supervision and mentorship visits at the health centres included clinical audits of charts with clinicians for all mothers who died or had significant morbidities. The objective of the audit was to determine the causes and assess the factors that contributed to the maternal deaths based on the “three delays model” [14]. The level of delay was determined for each case with a purpose of developing the action plans for intervention. Based on the three delays model the following were explored:

Delay in deciding to seek care: This included delay in seeking treatment at any time during antenatal care, up to and including the intrapartum period. The use of local herbs during labour and use of a traditional birth attendant before coming to the facility was also reviewed. Delay in arriving at a functional health facility: Any delay in transport from home once a decision had been made. Delay providing adequate care: This included any delay in referring from a facility that could not provide emergency obstetric care (e.g., a dispensary) or any delay in receiving adequate care at the receiving facility.

The maternal mortality audit allowed the team to assess the quality of care and decision making in order to improve care. The audit team was composed of an obstetrician, an assistant medical officer with anaesthesiology training, a paediatrician, care providers working at the supported HCs and senior midwives from the regional and council health management teams.

### Data collection tool and sources

The data were collected using a mobile data collection app called CommCare. Data were collected from the logbooks for Health Management Information System (HMIS), operating theatre logbooks and individual case files, which included partographs. The key dependent variables included the number of deliveries, number and types of maternal morbidities (women with obstetric complications) and maternal deaths. Variables for maternal death audits were the causes of maternal deaths and level(s) of delay using the three delays model. The audit team reviewed the case notes and partographs to establish the duration of the complication before admission to ascertain the delay in seeking care or reaching the facility, if she sought care at the traditional birth attendant before coming to the health facility and the appropriateness of the treatment provided at the health facility. The appropriateness of management was judged by comparing it with the national management guidelines.

### Strategies for research uptake

Strategies for uptake of the educational and mentoring programs and sustainability of the interventions in these health centres have been described elsewhere [12].

### Analysis

Data were extracted from the server into Microsoft Excel and analyzed using Stata (version 15). Tests of proportion were performed to compare the incidences of morbidities and case fatality rates during the baseline and intervention periods. The level of significance was set at a *p*-value of 0.05. The annual number of expected births in each catchment population was calculated using crude birth rate (annual live births per 1000 population), multiplied by the population during the year of interest. Of these births, 15% were estimated to be complicated [4, 15]. The met need for emergency obstetric care was determined as a proportion of pregnant women expected to have complications who were admitted for treatment [16].

## Results

### Proportion of expected births delivering in CEmONC facilities

Only two (Gairo and St. Joseph) of the five ASDIT project-supported health centres provided CEmONC services before the intervention period. Overall catchment population of the project supported health centres only increased by 17% from 101,983 in 2015 to 118,876 in 2019, while the proportions of all births in these facilities increased from 62% (2127/3417) in 2015 to 155% (5814/3757) in 2019 (Table 1). Following intervention, health centres revealed almost a similar picture of increased proportions of facility births in all categories of funding and governance models. The overall catchment population of the control health centres increased by 16% from 52,586 in 2015 to 60,861 in 2019, and the proportions of expected births delivering in these facilities increased from 158% (2791/1762) in 2015 to 230% (4419/1923) in 2019. Following introduction and improvement of CEmONC services, intervention facilities received a substantial number of women in labour from outside catchment population resulting in an increased proportion of deliveries at the health facility of more than 100% of the expected in four (80%) health centres.

**Table 1:** Proportions of expected births delivering in the health centres in intervention and control catchment populations before (2015) and after the intervention (2019)

Health Centre	2015			2019		
	Catchment population	Expected births^a^	Number and proportions of all births in the HC	Catchment population	Expected births	Number and proportions of all births in the HC
Intervention HCs						
St. Joseph	33,569	1125	559 (50%)	40,027	1265	2069 (164%)
Ngerengere	14,000	469	282 (60%)	20,000	632	438 (69%)
Kibati	8900	298	232 (78%)	10,183	322	585 (182%)
Melela	9542	320	162 (51%)	8649	273	334 (122%)
Gairo^b^	35,972	1205	892 (74%)	40,017	1265	2388 (189%)
Total	101,983	3417	2127 (62%)	118,876	3757	5814 (155%)
Control HCs						
Mkamba	7586	254	892 (351%)	8861	280	1146 (409%)
Mlimba	45,000	1508	1899 (126%)	52,000	1643	3273 (199%)
Total	52,586	1762	2791 (158%)	60,861	1923	4419 (230%)

^a^*Note*: expected births were computed from the catchment populations using the crude birth rates of 33.5 births per 1000 population in 2015 and 31.6 births per 1000 population in 2019 in Morogoro region [17]. ^b^Gairo catchment population is based on the projections of Gairo ward from the 2012 National Census [18]

### Met need for emergency obstetric care

As health centres began keeping pregnant women with obstetric complications rather than transferring them, it was expected that more maternal morbidity would be observed and documented. In addition, women with obstetric complications from the satellite dispensaries and beyond increasingly sought care from these facilities. The overall prevalence of maternal morbidities increased significantly from 10.5% (95% CI 9.6–11.4%) at baseline to 15.6% (95% CI 14.9–16.2%) during the intervention period in the intervention health facilities (Table 2). On the other hand, the overall prevalence of maternal morbidities was significantly lower in the control group and did not change in the control health centres, where it was 4.9% (95%CI 4.4–5.5%) at baseline and 5.9% (95% CI 5.5–6.4%) during the intervention period. The estimate of the proportion of all women with major obstetric complications who were treated at the intervention HCs (met need for emergency obstetric care) increased significantly from 45% (459/1025) at baseline (Jul 2014 – June 2016) to 119% (2010/1691) during the intervention period (Jul 2016 – June 2019) (Table 3). The met need for emergency obstetric care in the control group also increased from 53% (95% CI 49–58%) to 77% (95% CI 74–80%). The leading morbidities were obstructed labour, complications of abortions and postpartum haemorrhage in the intervention facilities contributing to 81% of the morbidities during the intervention period.

**Table 2:** Prevalence of maternal morbidities in the intervention and control facilities before and after intervention

Deliveries/	Intervention HCs		Control HCs	
Morbidity	Baseline	Intervention period	Baseline	Intervention period
	*n* (%)	*n* (%)	*n* (%)	*n* (%)
Obstructed labour	169 (3.8)	445 (3.4)	144 (2.5)	311 (2.8)
Postpartum haemorrhage	46 (1.0)	214 (1.7)	20 (0.4)	69 (0.6)
Antepartum haemorrhage	20 (0.5)	72 (0.6)	33 (0.6)	36 (0.3)
Pre eclampsia	29 (0.7)	97 (0.7)	35 (0.6)	111 (1.0)
Eclampsia	32 (0.7)	121 (0.9)	29 (0.5)	60 (0.5)
Ruptured uterus	8 (0.2)	53 (0.4)	8 (0.1)	12 (0.1)
Abortion complications	155 (3.5)	968 (7.6)	13 (0.2)	58 (0.5)
Others	0 (0.0)	40 (0.3)	0 (0.0)	7 (0.1)
Total morbidities	459 (10.5)	2010 (15.6)	282 (4.9)	664 (5.9)
Total deliveries	4392	12,918	5709	11,233

*Note*: *HCs* health centres; Baseline = Jul 2014 – June 2016; and intervention period = Jul 2016 – June 2019

**Table 3:** Met need for EmONC services and case fatality rate before and after the intervention in the control and intervention health centres

	Total deliveries	Maternal deaths	Maternal morbidities	Expected women with obstetric complications	Met need for EmONC	Case fatality rate	95% CI
Intervention HCs							
Baseline	4392	7	459	1025	45%	1.5%	0.6–3.1
Intervention period	12,918	22	2010	1691	119%	1.1%	0.7–1.6
Control HCs							
Baseline	5709	6	282	529	53%	3.3%	1.2–7.0
Intervention period	11,233	5	664	865	77%	0.8%	0.2–1.7

*Note*: Baseline = Jul 2014 – June 2016 and intervention period = Jul 2016 – June 2019; Expected women with obstetric complications was estimated at 15% of the expected births in the catchment populations [4, 15]

### Maternal deaths in the intervention facilities: factors and causes

Forty maternal deaths were registered during the baseline and intervention periods in the control and intervention health centres. Three quarters (30) of the deaths occurred at Gairo, Dumila and Mlimba health centres. Following intervention a similar picture of increased proportions of facility births in all categories of funding and governance models was noted. The proportion of women admitted to the health centres with obstetric complications, plus those who developed such complications after admission, and died before discharge (case fatality rate) decreased slightly from 1.5% (95% CI 0.6–3.1%) at baseline to 1.1% (95% CI 0.7–1.6%) during the intervention period in the intervention group and from 3.3% (95% CI 1.2–7.0%) to 0.8% (95% CI 0.2–1.7%) in the control group.

**Fig. 2:**
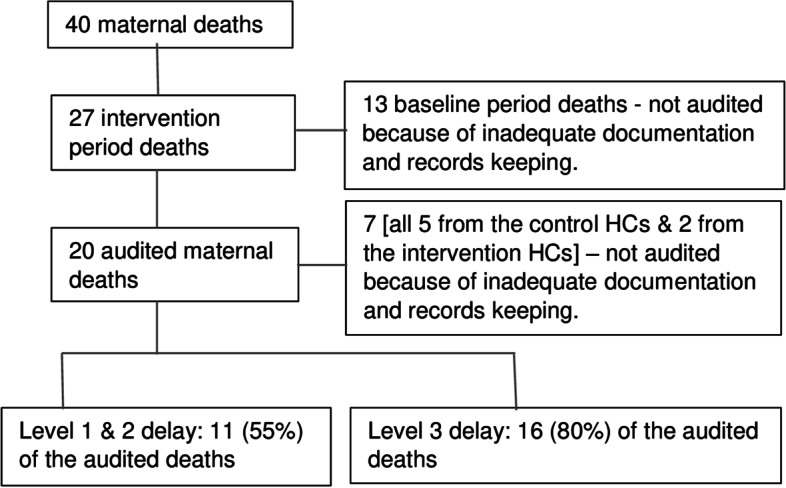
Flow diagram of the process of including maternal deaths for audit and causal factors identification

Thirteen (13) deaths that occurred before the intervention (i.e., at baseline) were not audited because of inadequate record-keeping (Fig. 2). The leading causes of maternal deaths that occurred during the intervention period were postpartum haemorrhage with 8 deaths (35%) followed by pre/ eclampsia which resulted in 5 deaths (22%). Other causes were puerperal sepsis 3 (13%), complications of anaesthesia 2 (4%), uterine rupture 2 (9%), severe anaemia 2 (9%) and antepartum haemorrhage 1 (4%). The causes of deaths were not established in four cases because of inadequate documentation and record keeping. Using the three delays model the level of delay could be determined in only 74% i.e., 20 cases out of 27 deaths that occurred during the intervention period. Level three delay was identified in 80% i.e., 16 of 20 whose case files had adequate information to judge the level of delay. It was difficult to establish the exact level of delay during audit in order to determine whether the delay was in seeking treatment or in reaching the facility if the delay was outside the facility. A delay in seeking treatment/ reaching the facility was found in 55% i.e., 11 out of 20 deaths including 3 women with postpartum haemorrhage and 2 with puerperal sepsis who were brought to the health facilities following delivery by traditional birth attendants. Delay in receiving appropriate treatment at the facility was found in 70% i.e., 14 out of 20 deaths and this was secondary to inadequate skills, inadequate supplies particularly blood for transfusion and an ineffective referral system.

## Discussion

Introduction and strengthening of CEmONC services in the health centres resulted in increased proportions of expected births delivering in CEmONC facilities and met need for emergency obstetric care, and reduction of direct obstetric case fatality rate. The met need for emergency obstetric care increased significantly from 45% at baseline to 119% during the intervention period in the intervention group, and increased from 53% (95% CI 49–58%) to 77% (95% CI 74–80%) in the control group.

### Proportion of expected births delivering in CEmONC facilities

Improving access to emergency obstetric care was achieved by introducing CEmONC services in underserved rural areas. Training of care providers was coupled with adoption of task sharing and supportive supervision along with continuous mentorship; and was supported by the presence of a national policy of free CEmONC services that promotes client utilization. Proportions of all births in almost all intervention and control facilities were more than 100% during the intervention period suggesting that almost all births from the catchment populations and others from outside took place at the CEmONC facilities. This figure is higher than the proportion of all births at the health facilities in Tanzania which is estimated at 77% [19]. Although giving birth at an emergency obstetric care facility does not equate with the quality of services it is a crude indicator of the use of obstetric services by pregnant women and a key factor in reducing maternal and perinatal mortality and morbidity [4, 20]. The fact that the proportions of all births in these facilities exceeded 100% suggests that many women came from outside the catchment population for delivery services. These findings suggest improved trust of surrounding community with the quality of services provided in these facilities. This sheds light on the need to expand CEmONC services in other rural facilities in order to reduce high workload in the existing comprehensive EmONC facilities.

### Met need for emergency obstetric care

Increased proportion of all women with major obstetric complications who were treated at the intervention HCs from 45% at baseline to 119% during the intervention period strongly suggests that a larger proportion of women who really needed life-saving obstetric care in the catchment populations received it. Studies indicate that met need for emergency obstetric care is inversely correlated with maternal mortality ratio [15]. The met need exceeded 100% in the intervention HCs probably because the facilities received women with obstetric complications from satellite dispensaries. It is also possible that the operational definitions of major direct obstetric complications were not properly adhered to [4], and that the fraction of 15% set for estimating women who develop obstetric complications is smaller in this setting.

### Case fatality rate

Although the direct obstetric case fatality rate was slightly lower than 2–10% reported in most low income countries it was substantially higher than the rates reported in high income countries such as the US (0.06% in 2000) [4, 21]. The major factor contributing to these deaths during the intervention period was delay in receiving appropriate treatment at the facility either because of inadequate skills, inadequate supplies particularly blood for transfusion and/or an ineffective referral system. As reported in other studies an incompetent health workforce is one of the leading factors crippling maternal health care in resource limited settings [22]. It has been reported that, in Tanzania, health care providers working in rural areas can correctly diagnose only 40% of common conditions [23]. All these findings suggest sub-optimal pre-service training standards and need for intervention at this level and in-service.

The deaths attributed to delay in reaching the CEmONC facility and those delayed at lower health facilities because of lack of transport suggest suboptimal arrangements of transport at individual and lower health facility levels. These findings reflect an ineffective obstetric emergency referral transport system in underserved areas in Tanzania. Efforts geared at strengthening CEmONC services in rural areas should also address emergency referral transport system including encouraging pregnant women to make arrangements with local owners of transport as part of birth preparedness. Availability of timely and effective emergency referral transport is an essential component of any health system and is crucial to achieving high quality emergency obstetric and newborn care [24–26]. Arrangements of transport enable timely transportation of critically ill patients to higher level facilities which are better equipped and have the expertise required to manage complicated cases. Transferring a higher proportion of women with obstetric complications in the control than the intervention facilities could partly explain a substantial reduction of case fatality rate in the control facilities. It is also possible that the trained teams in the intervention facilities were over-confident and did not transfer critically ill patients requiring higher level of care. However, this was not captured during the audit of the deaths.

### Sustainability and scalability of the intervention

The educational and mentoring programs presented in this study warrant scale up because they: 1) address a problem of public health importance in Tanzania and other countries in the region; 2) are remarkably effective with minimal harm; 3) build on the available resources, i.e., the workforce for health and infrastructure; 4) are consistent with and supported by the government policy and priorities – to increase the number of HCs providing CEmONC services from 12 to 50% [5]; and there is evidence that the intervention is feasible in countries with similar constraints to Tanzania on health system resources [27, 28].

### Limitations

Both groups (intervention and control facilities) were in Morogoro region. Therefore, regular updates of the ASDIT project implementation to the regional health management teams could have influenced uptake of similar interventions to the control group facilities. Also, there was considerable under-documentation of individual case files for maternal deaths and inefficient record-keeping systems before the intervention in the intervention group, and before and after intervention in the control group. This prevented a more in-depth analysis of why maternal deaths occurred especially in the control centres. During the intervention period the project supported the intervention health facilities with stationeries, theatre logbooks and trained care providers on the importance of proper documentation, medical records keeping and utilization of data in order to improve management of health records. The control facilities did not receive such support.

## Conclusions

Findings from this study indicate that if CEmONC services are made available more widely, the met need for obstetric complications increases. However, many women and their neonates still lose their lives even after reaching the health facilities because of an inadequately-trained health workforce, lack of essential medical supplies including blood transfusion services and an ineffective referral system. The effort to scale up CEmONC services in underserved rural areas in sub-Saharan Africa should be accompanied by strategies for strengthening skills for CEmONC for in-service and pre-service training programs and the referral system particularly from the primary health facilities.

## Data Availability

The datasets generated and/or analysed during the current study are not publicly available due to government restrictions on sharing data but are available from the corresponding author on reasonable request.
